# Ocular perfusion pressure and ophthalmic artery flow in patients with normal tension glaucoma

**DOI:** 10.1186/s12886-016-0215-3

**Published:** 2016-04-14

**Authors:** Amir Samsudin, Nadine Isaacs, Mei-Ling Sharon Tai, Norlina Ramli, Zahari Mimiwati, May May Choo

**Affiliations:** Department of Ophthalmology, University of Malaya Eye Research Centre, University of Malaya, Kuala Lumpur, Malaysia; Division of Neurology, Department of Medicine, University of Malaya, Kuala Lumpur, Malaysia

## Abstract

**Background:**

Vascular insufficiency has been reported to be a cause of normal tension glaucoma (NTG). The aim of this study was to compare ocular perfusion pressure (OPP) and ophthalmic artery flow (OAF) between patients with NTG and those without glaucoma.

**Methods:**

We considered one eye each from 31 NTG and 15 non-glaucoma control patients. Blood pressure and intraocular pressure (IOP) were measured in the sitting position, for calculation of OPP. Humphrey visual field (HVF) assessment was then carried out on NTG patients. All patients then underwent Transcranial Doppler ultrasound measurements of OAF parameters, including mean flow velocity (MFV), end diastolic velocity (EDV), peak systolic velocity (PSV) and resistive index (RI). We looked at differences in OPP and OAF parameters between the two groups, and their correlations in NTG patients. T-tests, *χ*^2^, ANOVA and Pearson Correlation tests were performed, with *p* < 0.05 considered statistically significant.

**Results:**

There were no statistically significant differences in OPP between the NTG and control groups (60.5+/-8.7 mmHg and 62.9+/-10.2 mmHg respectively, *p* = 0.393), and also no statistically significant differences in MFV, EDV, PSV and RI (all *p* > 0.05). In the NTG group, there were positive correlations between OPP and both MFV (*r* = 0.416, *p* = 0.020) and EDV (*r* = 0.369, *p* = 0.041). There were no statistically significant correlations between HVF mean deviation and OPP or OAF parameters (all *p* > 0.05).

**Conclusion:**

There is no difference in OPP and OAF parameters between patients with NTG and non-glaucoma controls, suggesting that vascular insufficiency or dysregulation by themselves may not account for the pathogenesis of NTG.

## Background

Normal tension glaucoma (NTG) is a variant of primary open angle glaucoma (POAG). It is characterized by glaucomatous optic disc damage and visual field loss; intraocular pressure (IOP) of ≤21 mmHg; open drainage angles on gonioscopy; and absence of secondary causes for the glaucomatous optic disc damage. The pathogenesis of NTG has not been conclusively determined, although vascular insufficiency and decreased optic disc resistance have been postulated [1]. The former has attracted the interest of a number of researchers. One study compared intraocular circulatory dynamics and visual field defects between primary open angle glaucoma and normal tension glaucoma. It concluded that the vascular resistance of the ophthalmic artery could be associated with the development of visual field defects in NTG patients, as opposed to patients with POAG [2]. Another study evaluated blood flow in patients with POAG and NTG, and found that blood flow in the ophthalmic artery, central retinal artery and posterior ciliary arteries were reduced in both conditions when compared to controls [3].

Ocular perfusion pressure (OPP) is the calculated difference between mean arterial blood pressure and IOP. It is an important parameter which determines the perfusion of the optic nerve head. OPP has been linked with glaucoma in many epidemiologic studies [4–8]. In particular, low OPP has been found to be a risk factor for the development of glaucoma. Both high IOP and low systemic blood pressure can lead to low OPP [9, 10], which in turn may lead to reduced ocular blood flow and ischemia of the optic nerve head. In this study, ocular perfusion pressure is calculated as follows:

$$ \mathrm{O}\mathrm{P}\mathrm{P} = 2/3\ \left[\mathrm{D}\mathrm{B}\mathrm{P} + 1/3\ \left(\mathrm{S}\mathrm{B}\mathrm{P}-\mathrm{D}\mathrm{B}\mathrm{P}\right)\right]\ \hbox{--}\ \mathrm{I}\mathrm{O}\mathrm{P}, $$

where DBP is diastolic blood pressure and SBP is systolic blood pressure.

The ophthalmic artery is the first branch of the internal carotid artery distal to the cavernous sinus. Branches of the ophthalmic artery supply all the structures in the orbit. The ophthalmic artery can be detected by Transcranial Doppler (TCD) ultrasound. This modality measures the velocity of blood flow through the blood vessels in the brain. The velocities follow the cardiac cycle, and measurements of the peak systolic velocity (PSV) and mean flow velocity (MFV) are usually taken. End diastolic velocity (EDV) is usually calculated from

$$ \mathrm{E}\mathrm{D}\mathrm{V} = \left(\left[3\ \mathrm{M}\mathrm{F}\mathrm{V}\right] - \mathrm{P}\mathrm{S}\mathrm{V}\right)/2. $$

The Resistive Index (RI) is a ratio used to relate the systolic and diastolic velocities to each other. It is relatively operator independent, and is a good method to quantify the vascular resistance of the circulation [11]. RI reflects the resistance to blood flow distal to the site of measurement. It primarily represents a quantification of end-organ resistance, in this case of the low-resistance vascular beds in the cerebrovascular region [12].

The main objectives in this study were to look for differences in OAF and OPP between NTG and non-glaucomatous patient, and to determine the correlation between OPP and OAF in NTG patients. We also aimed to identify if OAF and OPP parameters changed with increasing severity of glaucoma in NTG patients.

## Methods

This was a cross-sectional, non-interventional study at the Ophthalmology clinic and Neurology laboratory of the University Malaya Medical Centre, Kuala Lumpur. Data was collected from August till December 2014. This study complied with the tenets of the Declaration of Helsinki and was approved by the Medical Ethics Committee of the University Malaya Medical Centre. Informed consent was given by every subject prior to their enrolment.

NTG patients were defined as those having glaucomatous visual field defects corresponding to optic disc changes; IOP of ≤21 mmHg on 24-h phasing; open iridocorneal angles on gonioscopy; and no evidence of secondary glaucoma such as pseudoexfoliation or pigment dispersion. Exclusion criteria included presence of diabetic or hypertensive retinopathy, past history of cerebrovascular accidents which could contribute to visual field defects, and eyes which had undergone any kind of glaucoma filtration or drainage device surgery. Controls were selected from patients attending the Ophthalmology clinic for various conditions, including cataract and diabetic retinopathy screening. These patients had cup-disc ratios within normal limits and IOP of ≤21 mmHg.

All subjects underwent full eye examinations including visual acuity, slit lamp examination, and IOP measurement. IOP measurements were taken using a Goldmann applanation tonometer (Haag-Streit, Koeniz, Switzerland). Two readings were taken and the average recorded. Gonioscopy was carried out if a prior examination had not been done in the last 6 months. Dilated fundoscopy was done, and the cup-disc ratios assessed clinically. Following this, blood pressure measurements were taken in the sitting position using an automated blood pressure monitor (TM-2540R; A&D, Tokyo, Japan). SITA-standard, central 24-2 Humphrey visual field assessment was then carried out for the NTG patients. Reliable results were defined as false positive and false negative values of <33 %, and the Mean Deviation (MD) value was taken as a measure of glaucoma severity. Finally, all subjects had Transcranial Colour Doppler imaging of the OAF at the Neurology laboratory, using a SONARA Transcranial Doppler system (Natus Medical Inc., Pleasanton, USA). One experienced technician performed all the examinations. The technique was standardised; with the patient lying supine and head resting on one pillow, the transducer was placed on the closed eyelid, with coupling gel in between. No overt force was used during placement of the transducer. Measurements took about 5 min to obtain per eye. MFV, PSV and RI were measured while EDV was calculated based on the formula mentioned previously. All these procedures were performed between 11 am and 1 pm on the same day.

We analysed the right eyes of the patients and controls, except in one patient who had previously undergone a filtration procedure in that eye. In this patient, the left eye was analysed. Data analysis was performed using SPSS version 14 (SPSS Inc., Chicago, USA). T-tests, *χ*^2^ tests and ANOVA were performed to test differences between the groups. Pearson correlation tests were performed to study the correlation between variables in the NTG group. *P* < 0.05 was considered statistically significant.

## Results

The study group consisted of 31 NTG patients and 15 normal patients. There were no statistically significant differences in demographics, OPP parameters, and OAF parameters between the NTG and control groups (Table 1). Table 2 shows the characteristics of our NTG patients divided into mild, moderate and severe categories according to their MD values. Table 3 shows the correlations between OPP, OAF parameters and MD in the NTG group. OPP correlated with MFV and EDV. MFV correlated with PSV and EDV. EDV correlated negatively with RI. There was no statistically significant correlation between MD and OPP or OAF parameters, indicating no correlation with glaucoma severity.

**Table 1:** Group demographics, OPP parameters, and OAF parameters in NTG and control groups

Variable	Group		*P* value
	NTG (*n* = 31)	Normal (*n* = 15)	
Age (years)	66.3 +/- 9.2	65.4 +/- 8.1	0.512
Gender, male	16 (52 %)	6 (40 %)	0.525
female	15 (48 %)	9 (60 %)	
Presence of Diabetes Mellitus	14 (45 %)	10 (67 %)	0.214
Presence of Hypertension	17 (55 %)	11 (73 %)	0.332
Duration of NTG (years)	4.6 +/- 2.8	N/A	
Mean Deviation, MD (dB)	-7.82 +/- 6.69	N/A	
IOP (mmHg)	11.2 +/- 2.6	11.1 +/- 2.1	0.904
Systolic BP (mmHg)	149.9 +/- 23.8	152.1 +/- 17.4	0.752
Diastolic BP (mmHg)	85.9 +/- 8.8	90.6 +/- 14.4	0.174
Ocular Perfusion Pressure, OPP (mmHg)	60.5 +/- 8.7	62.9 +/- 10.2	0.393
Mean Flow Velocity, MFV (cm/s)	16.99 +/- 3.40	16.38 +/- 2.94	0.557
Peak Systolic Velocity, PSV (cm/s)	35.48 +/- 9.07	32.76 +/- 10.41	0.369
End Diastolic Velocity, EDV (cm/s)	7.74 +/- 3.32	8.19 +/- 3.64	0.679
Resistive Index, RI	0.77 +/- 0.10	0.73 +/- 0.12	0.295

Results shown as mean +/- SD

**Table 2:** Characteristics of NTG patients divided into mild, moderate and severe groups

	Mild	Moderate	Severe	*P* value
	(MD < -6 dB)	(MD -6 to -12 dB)	(MD > -12 dB)	
n	16 (51.6 %)	8 (25.8 %)	7 (22.6 %)	
Mean Deviation, MD (dB)	-3.00 +/- 1.55	-8.59 +/- 2.20	-17.96 +/- 5.42	<0.001
Cup:Disc Ratio, CDR	0.69 +/- 0.11	0.75 +/- 0.09	0.80 +/- 0.08	0.076
Duration (years)	5.2 +/- 3.5	3.7 +/- 2.1	4.2 +/- 1.0	0.486
Number of medications (units)	1.3 +/- 0.4	1.6 +/- 0.5	1.9 +/- 0.9	0.072
Ocular Perfusion Pressure, OPP (mmHg)	66.00 +/- 10.78	59.06 +/- 7.42	57.29 +/- 6.62	0.096
Mean Flow Velocity, MFV (cm/s)	17.43 +/- 2.00	17.06 +/- 4.01	16.33 +/- 3.49	0.828
Peak Systolic Velocity, PSV (cm/s)	32.23 +/- 6.91	36.07 +/- 10.74	37.84 +/- 6.79	0.471
End Diastolic Velocity, EDV (cm/s)	10.03 +/- 1.32	7.55 +/- 3.57	5.57 +/- 2.94	0.027*
Resistive Index, RI	0.70 +/- 0.10	0.78 +/- 0.12	0.83 +/- 0.06	0.064

Results shown as mean +/- SD. *indicates statistically significant difference between mild and severe groups

**Table 3:** Correlations between OPP, OAF parameters and MD in NTG group

	Mean flow velocity	Peak systolic velocity	End diastolic velocity	Resistive index	Mean deviation (MD)
Ocular Perfusion					
Pressure, OPP					
*r* value	0.416	0.198	0.369	0.006	-0.027
*p* value	0.020	0.286	0.041	0.975	0.884
Mean flow					
Velocity, MFV					
*r* value		0.769	0.485	-0.044	-0.002
*p* value		<0.001	0.006	0.815	0.989
Peak systolic					
velocity, PSV					
*r* value			-0.186	0.524	-0.167
*p* value			0.317	0.062	0.368
End diastolic					
Velocity, EDV					
*r* value				-0.784	0.225
*p* value				<0.001	0.223
Resistive index, RI					
*r* value					-0.171
*p* value					0.357

## Discussion

This study looked for differences in OPP and OAF parameters between NTG patients and non-glaucoma controls. We found no difference in OPP between the two groups. Similarly, Ramli et al. found no difference in OPP between patients with NTG and controls when OPP was measured during the day [13]. However, they found that OPP measured at night showed a significant difference between the groups, being lower in NTG patients. Sung et al. compared progressing and non-progressing NTG patients and found that the progressing group had wider 24-h fluctuations in mean arterial pressure and mean OPP [14]. We thus believe that day and night differences, or fluctuations in OPP may be more important in the development or progression of NTG.

There were also no differences in the ophthalmic artery flow parameters (MFV, PSV, EDV and RI) between the groups. Plange et al. found that patients with NTG had lower PSV and EDV when compared to healthy controls [15]. Kaiser et al. and Butt et al. found lower EDV in NTG patients when compared to healthy controls, although there were no differences in PSV [3, 16]. However, Bossuyt et al. more recently reported that vascular dysregulation in NTG was not affected by structure and function of the microcirculation or macrocirculation, including arterial stiffness, total peripheral resistance, cardiac output, and both peripheral and central haemodynamics at rest [17]. They concluded that provocation tests may be needed to reveal alterations in cardiovascular function in NTG patients. This reason may contribute to why our non-provocative study also did not show any differences between the groups.

We found moderate positive correlations between OPP and both MFV and EDV in the NTG patients. In our literature search, we found only one other study which looked into this relationship. Gherghel et al. found a positive correlation between OPP and EDV in patients with progressive POAG and NTG [10]. However, they found that the correlation was not significant in ‘stable’ glaucoma and normal patients. They concluded by stating that alterations in ocular blood flow may contribute to the progression of glaucomatous damage. We also found that MFV correlated with PSV and EDV. This may not be unexpected, as MFV as a mean value is related to the peak value that is PSV. Additionally, as EDV is a function of both MFV and PSV, it is also expected to correlate with the two values. EDV correlated negatively with the RI, which is similar to that found by Harris et al., who noted that patients with NTG exhibited a low EDV and a high RI in the ophthalmic artery when compared with controls [18]. Rankin et al. showed that in both chronic open-angle glaucoma and NTG, there is a significant increase in RI and a decrease in the mean blood velocity in both the central retinal artery and short posterior ciliary arteries [12]. These findings can also be expected; in all blood vessels, flow is directly proportional to the pressure difference along the vessel, and is inversely proportional to the resistance of the vessel [19].

No statistically significant correlation was found between severity of glaucoma, as evidenced by MD values, and either OPP or OAF parameters. We believe that this may have been contributed by the fact that our study had more patients with mild glaucoma (MD < -6 dB, 51.6 %) than those with moderate (MD -6 to -12 dB, 25.8 %) or severe (MD > -12 dB, 22.6 %) glaucoma. In a study conducted by Kondo et al., they noted that NTG patients with less severe glaucoma had significantly better retrobulbar haemodynamics compared to those with more severe glaucoma [20]. In our case, although the patterns in OPP, MFV, EDV and RI supported the authors’ findings, only the difference in EDV was of statistical significance (Table 2).

There are a few limitations to this study. We measured blood flow velocity in the ophthalmic artery as an indicator of the optic nerve microcirculation. As the artery supplies all structures in the orbit, the non-statistically significant changes and correlations observed in this study do not rule out changes in arteries directly supplying the optic nerve such as the posterior ciliary arteries. Next, there was no washout of the patients’ anti-glaucoma medications. Some of the patients had advanced glaucoma and it was deemed unsuitable to stop treatment for this group. Had we found any differences between the groups, it would be difficult to attribute them to the glaucoma, or to the anti-glaucoma medication. We also did not exclude patients with underlying diabetes mellitus or hypertension. Although it was assumed that these patients did not have severe microvascular changes based on the fact that they did not have diabetic or hypertensive retinopathy, there may have been underlying milder microvascular damage in the eyes of both groups of subjects, which could have influenced the outcome of this study. Finally, the sample size was also relatively small, and thus had limited power to detect differences between the groups. In future studies, a larger sample size may find differences between NTG patients and healthy controls that were not discovered in this study.

## Conclusion

There is no difference in OPP and OAF parameters between patients with NTG and non-glaucoma controls, suggesting that vascular insufficiency or dysregulation by themselves may not account for the pathogenesis of NTG.

### Ethics approval and consent to participate

This study complied with the tenets of the Declaration of Helsinki and was approved by the Medical Ethics Committee of the University Malaya Medical Centre. Informed consent was given by every subject prior to their enrolment.

### Consent for publication

Not applicable.

### Availability of data and materials

The first author has full access to all the data. This will be shared upon request.

## Abbreviations

ANOVA: Analysis of variance
BP: Blood pressure
CDR: Cup: disc ratio
DBP: Diastolic blood pressure
EDV: End diastolic velocity
HVF: Humphrey visual field
IOP: Intraocular pressure
MD: Mean deviation
MFV: Mean flow velocity
NTG: Normal tension glaucoma
OAF: Ophthalmic artery flow
OPP: Ocular perfusion pressure
POAG: Primary open angle glaucoma
PSV: Peak systolic velocity
RI: Resistive index
SBP: Systolic blood pressure
TCD: Transcranial Doppler (ultrasound)

## References

[1] Mozaffarieh M, Flammer J (2013). New insights in the pathogenesis and treatment of normal tension glaucoma. Curr Opin Pharmacol.

[2] Yamazaki Y, Hayamizu F (1995). Comparison of flow velocity of ophthalmic artery between primary open angle glaucoma and normal tension glaucoma. Br J Ophthalmol.

[3] Kaiser HJ, Schoetzau A, Stumpfig D, Flammer J (1997). Blood flow velocities of the extraocular vessels in patients with high tension and normal tension primary open angle glaucoma. Am J Ophthalmol.

[4] Tielsch JM, Katz J, Singh K, Quigley HA, Gottsch JD, Javitt J (1991). A population-based evaluation of glaucoma screening: The Baltimore Eye Study. Am J Epidemiol.

[5] Hoffman A, Breteler MB, van Duijn CM, Krestin GP, Pols HA, Stricker BHC (2007). The Rotterdam study: objectives and study design. Eur J Epidemiol.

[6] Bonomi L, Marchini G, Marraffa M, Bernardi P, Morbio R, Varotto A (2007). Vascular risk factors for primary open angle glaucoma: the Egna-Neumarkt study. Ophthalmology.

[7] Leske MC, Wu SY, Hennis A, Honkanen R, Nemesure B, BESs Study Group (2008). Risk factors for incident open-angle glaucoma: the Barbados Eye Studies. Ophthalmology.

[8] Leske MC, Heijl A, Hyman L, Bengtsson B, Dong L, Yang Z (2007). Predictors of long term progression in the early manifest glaucoma trial. Ophthalmology.

[9] Leske MC (2009). Ocular perfusion pressure and glaucoma: clinical trial and epidemiologic findings. Curr Opin Ophthalmol.

[10] Gherghel D, Orgul S, Gugleta K, Gekkieva M, Flammer J (2000). Relationship between ocular perfusion pressure and retrobulbar blood flow in patients with glaucoma with progressive damage. Am J Ophthalmol.

[11] Rankin SJ (1999). Colour Doppler imaging of the retrobulbar circulation in glaucoma. Surv Ophthalmol.

[12] Rankin SJ, Walman BE, Buckley AR, Drance SM (1994). Color Doppler imaging and spectral analysis of the optic nerve vasculature in glaucoma. Am J Ophthalmol.

[13] Ramli N, Nurull BS, Noran NN, Mimiwati Z (2013). Low nocturnal ocular perfusion pressure as a risk factor for normal tension glaucoma. Prev Med.

[14] Sung KR, Lee S, Park SB, Choi J, Kim ST, Yun SC (2009). Twenty-four hour ocular perfusion pressure fluctuation and risk of normal-tension glaucoma progression. Invest Ophthalmol Vis Sci.

[15] Plange N, Kaup M, Weber A, Harris A, Arend KO, Remky A (2009). Performance of colour Doppler imaging discriminating normal tension glaucoma from healthy eyes. Eye.

[16] Butt Z, McKillop G, O’Brien C, Allan P, Aspinall P (1995). Measurement of ocular blood flow velocity using colour Doppler imaging in low tension glaucoma. Eye.

[17] Bossuyt J, Vendekerckhove V, De Backer TL, Van de Velde S, Azermai M, Stevens AM (2015). Vascular dysregulation in normal-tension glaucoma is not affected by structure and function of the microcirculation or macrocirculation at rest. Medicine (Baltimore).

[18] Harris A, Sergott RC, Spaeth GL, Katz JL, Shoemaker JA, Martin BJ (1994). Color Doppler analysis of ocular vessel blood velocity in normal-tension glaucoma. Am J Ophthalmol.

[19] Klabunde RE (2011). Cardiovascular physiology concepts.

[20] Kondo Y, Niwa Y, Yamamoto T, Sawada A, Harris A, Kitazawa Y (2000). Retrobulbar hemodynamics in normal-tension glaucoma with asymmetric visual field change and asymmetric ocular perfusion pressure. Am J Ophthalmol.

